# Identification and characterization of the novel nuclease activity of human phospholipid scramblase 1

**DOI:** 10.1186/s12858-016-0067-8

**Published:** 2016-05-20

**Authors:** Ulaganathan Sivagnanam, Shweta Narayana Murthy, Sathyanarayana N. Gummadi

**Affiliations:** From the Applied and Industrial Microbiology Laboratory, Department of Biotechnology, Bhupat and Jyoti Mehta School of Biosciences, Indian Institute of Technology Madras, Chennai, 600 036 India

**Keywords:** Nuclease, Histidine, Kunitz assay, Mg^2+^ dependent, Site-directed mutagenesis, Scramblase

## Abstract

**Background:**

Human phospholipid scramblase 1 (hPLSCR1) was initially identified as a Ca^2+^ dependent phospholipid translocator involved in disrupting membrane asymmetry. Recent reports revealed that hPLSCR1 acts as a multifunctional signaling molecule rather than functioning as scramblase. hPLSCR1 is overexpressed in a variety of tumor cells and is known to interact with a number of protein molecules implying diverse functions.

**Results:**

In this study, the nuclease activity of recombinant hPLSCR1 and its biochemical properties have been determined. Point mutations were generated to identify the critical region responsible for the nuclease activity. Recombinant hPLSCR1 exhibits Mg^2+^ dependent nuclease activity with an optimum pH and temperature of 8.5 and 37 °C respectively. Experiments with amino acid modifying reagents revealed that histidine, cysteine and arginine residues were crucial for its function. hPLSCR1 has five histidine residues and point mutations of histidine residues to alanine in hPLSCR1 resulted in 60 % loss in nuclease activity. Thus histidine residues could play a critical role in the nuclease activity of hPLSCR1.

**Conclusions:**

This is the first report on the novel nuclease activity of the multi-functional hPLSCR1. hPLSCR1 shows a metal dependent nuclease activity which could play a role in key cellular processes that needs to be further investigated.

## Background

Human phospholipid scramblases (hPLSCRs) are type II single pass transmembrane proteins involved in the Ca^2+^ dependent ATP independent translocation of phospholipids (PLs) across the lipid bilayer [1]. hPLSCR1 was first identified as a 37 kDa integral membrane protein from human erythrocytes, which was capable of bidirectional scrambling of PLs between two leaflets of the lipid bilayer when reconstituted into liposomes. The gene encoding hPLSCR1 was cloned, overexpressed and the recombinant hPLSCR1 exhibited low scrambling activity compared to that of erythrocyte PL scramblase [1]. Five homologs of scramblases (hPLSCR1–5) have been identified so far. hPLSCR1, 3 and 4 were expressed in wide variety of tissues; whereas hPLSCR2 was found only in testis and hPLSCR5 has been shown only at mRNA level [2, 3].

hPLSCR1 possesses a single transmembrane helix but can localize to both plasma membrane (PM) and nucleus. It has been reported that scramblases destroy the lipid asymmetry in PM when intracellular Ca^2+^ levels were elevated by 1000 fold [1]. Previous studies showed that phosphatidylserine (PS) exposure during apoptotic conditions was directly related to hPLSCR1 expression levels and the amount of PS exposed to the cell surface could be manipulated by modifying the expression levels of hPLSCR1 [4]. Subsequent reports have questioned the role of hPLSCR1 in PS exposure [5–9]. Recently, a member of Ca^2+^ dependent ion channels, TMEM16F, and members of Xkr protein family were identified to be responsible for phospholipid scrambling in plasma membrane [10–13]. These reports along with other unusual factors for PL translocators such as low molecular weight, single transmembrane domain and slow rate of PL scrambling in-vitro support the argument that hPLSCR1 may not be a true scramblase and can have varied roles within the cell apart from phospholipid scrambling.

hPLSCR1 is a multifunctional protein involved in several other cellular processes including cell signaling, cell proliferation, transcriptional regulation and antiviral defense [14–18]. Overexpression of hPLSCR1 in myeloid cells inhibited tumorigenesis and enhanced cell differentiation [19, 20]. hPLSCR1 interacts with variety of signaling molecules such as epidermal growth factor receptor, c-Abl, Src kinase, Shc and onzins [21–23]. hPLSCR1 also interacts with nuclear proteins including importin-α and topoisomerase II [24, 25] and was shown to exhibit high affinity to chromatin [26]. hPLSCR1 expression is strongly stimulated in response to interferons and viral infections [6, 14]. Interferons are well known to stimulate the expression of several nucleases as a part of antiviral defense [27]. hPLSCR1 inhibits Hepatitis B virus replication partly by activating the Jak/Stat pathway [28]. hPLSCR1 interacts with Human T-cell leukemia virus type-1 (HTLV-1) and represses the Tax-dependent transactivation during HTLV-1 infection [29].

hPLSCR1 is characterized by five distinct domains conserved across the species: (i) C- terminal helix that spans the plasma membrane and is responsible for activity; (ii) Cysteine palmitoylation motif which helps in anchoring the protein to the membrane; (iii) Nuclear localization signal which helps in nuclear transport; (iv) an EF hand like Ca^2+^ binding motif and (v) DNA binding domain (M^86^-E^118^) [3]. The EF hand like Ca^2+^ binding motif is vital for Ca^2+^ binding and scramblase activity of hPLSCR1. Mutations in this motif rendered the protein inactive as the protein lost its ability to bind to calcium [30]. In a previous study, we showed that apart from Ca^2+^ binding to the EF-hand like motif, hPLSCR1 could also bind to a variety of metal ions including Mg^2+^ [31]. The DNA binding domain was shown to bind with the promoter of inositol 3-phosphate receptor (IP3R), enhancing the expression of IP3R [32]. Recently we identified a unique N-terminal proline rich domain (PRD) in hPLSCR1 and showed that the PRD is essential for the oligomerization and functional activation of hPLSCR1 [33].

hPLSCR1 interacts with c-terminal domain (CTD) of topoisomerase IIα (topo IIα) and enhances its decatenation activity [25]. The mechanism of the enhancement of topo IIα catalytic activity by hPLSCR1 is not yet understood. We hypothesize that the mechanism of enhancement of the activity of topo IIα by hPLSCR1 could happen in two ways: (i) hPLSCR1 helps in unwinding or cleaving of catenated substrate for the topo IIα to subsequently decatenate; (ii) hPLSCR1 might have independent nuclease properties that could compliment the decatenation activity of topo IIα.

Based on the ability of hPLSCR1 to bind to DNA and Mg^2+^, cytoplasmic localization, stimulation by interferons, and enhancement of decatenation activity of topo IIα, we believed that hPLSCR1 could moonlight as a metal ion dependent nuclease. In this study, we have shown that recombinant hPLSCR1 exhibits nuclease properties contributing to a novel feature of this multifunctional protein.

## Methods

### Overexpression and purification of hPLSCR1

hPLSCR1 cDNA clone was obtained from Origene, MD, USA, and was subsequently cloned into pET-28a (+) bacterial expression vector with an N-terminal His tag. This was transformed into *E. coli* BL-21 (DE3) and grown in LB media containing kanamycin (50 mg/l). Overexpression and purification were performed as described earlier [34]. Briefly, cells were lysed in buffer (20 mM Tris–HCl (pH – 7.4), 200 mM NaCl) by sonication, hPLSCR1 formed inclusion bodies (IB). N- lauroylsarcosine (N-LS) was used to recover native protein from IB followed by dialysis to remove N-LS and the proteins were purified to homogeneity using Ni^2+^-NTA chromatography. The eluted fractions from Ni^2+^-NTA chromatography were subjected to anion-exchange chromatography where DEAE sepharose (GE Healthcare, LC, UK) was used. The NaCl eluted protein fractions were again loaded in Ni^2+^-NTA resin and eluted. The purified protein was further concentrated by Amicon centrifugal filters (10 kDa cut-off) (Millipore, MA, USA) and visualized by silver staining or coomassie staining and confirmed by western blotting using anti-hPLSCR1 monoclonal antibody (Name: PLSCR1 antibody 1E9; catalog no - sc59645; specificity -human; origin - mouse monoclonal IgG_1_, Santa Cruz Biotechnology, TX, USA).

### Generation of point mutant of hPLSCR1

Overlap PCR method was used to generate point mutations in hPLSCR1 where the 5 histidines (H12, H53, H111, H2111, H262) were mutated to alanine and was subsequently cloned in pET28a (+), confirmed by sequencing and named as Mut-hPLSCR1. Overexpression and purification of Mut-hPLSCR1 were done as described earlier. Briefly, mutagenic primers were synthesized to incorporate the specific point mutations (Fig. 8a). Using wild type hPLSCR1 gene as a template, PCR was performed with the following combinations of primers to produce 6 fragments and named as follows. Stage 1 PCR: Fragment 1 - F1, R6; Fragment 2 - F2, R5; Fragment 3 - F3, R4; Fragment 4 - F4, R3; Fragment 5 -F5, R2; Fragment 6 – F6, R1. Stage 2 PCR: Adjacent fragments are used as a template with appropriate primers as follows. Initial 10 cycles of PCR were performed without the primers after which the primers were added; Fragment A - Fragment 1 + Fragment 2 – Primers – F1 and R5; Fragment B - Fragment 3 + Fragment 4 – Primers – F3 and R3; Fragment C – Fragment 5 + Fragment 6 – Primers – F5 and R1; Stage 3 PCR: Fragment I – Fragment A + Fragment B – Primers F1 and R3; Fragment II – Fragment B + Fragment C – Primers F3 and R1. Stage 4 PCR: Mutant hPLSCR1 – Fragment I + Fragment II – Primers F1 and R1.

### Decatenation assay

Decatenation assay was performed using the topo II decatenation assay kit (Topogen, CO, USA) as per manufacturer’s instructions. Briefly, 200 ng of kinetoplast DNA (kDNA) in standard Topo assay buffer (50 mM Tris–HCl (pH - 8.0), 150 mM NaCl, 10 mM MgCl_2_ and 0.5 mM dithiothreitol) was treated with 1 U of topo IIα pretreated with hPLSCR1 (30 min, 4 °C) and incubated for 15 min at 37 °C. Negative control has only the kDNA but not the enzyme. The products were visualized on a 1 % agarose gel stained with ethidium bromide.

### Nuclease activity: gel assay

Nuclease assays were performed with 20 pmol of purified recombinant hPLSCR1, 200 ng of yeast/ human genomic DNA as substrate in the assay buffer containing 50 mM Tris–HCl (pH - 8.0), 150 mM NaCl, 10 mM MgCl_2_ and 0.5 mM dithiothreitol to a total volume of 20 μl and incubated at 37 °C for 1 h. The reaction products were separated by electrophoresis on 1 % agarose gel with ethidium bromide (0.5 μg/ml); 12 % native PAGE stained with ethidium bromide was also used to visualize the products of the nuclease reaction.

### Nuclease activity: Kunitz assay

Kunitz assays were performed with 1 pmol/μl of purified recombinant hPLSCR1, 50 μg/ml of calf thymus DNA (Sigma Aldrich, USA) as the substrate in a buffer containing 50 mM Tris–HCl (pH - 8.0), 150 mM NaCl, 10 mM MgCl_2_ and 0.5 mM dithiothreitol to a total volume of 500 μl and incubated at 37 °C for 15 min. Negative controls contained only the DNA substrate without hPLSCR1. The difference in A_260_ between control and sample was considered for measuring the nuclease activity. One Kunitz unit is defined as the amount of enzyme added to 1 mg/ml of DNA that causes an increase in absorbance of 0.001 per minute at 260 nm at 37 °C. The increase in absorbance is due to the release of free nucleotides upon degradation of polymerized DNA [35]. Briefly, the enzyme activity is quantified as follows.

- Amount of DNA : 25 μg (final concentration – 50 μg/ml)
- Dilution factor : 20
- Amount of protein : 1 pmol/μl
- Incubation time : 15 min
- Test : DNA treated with hPLSCR1
- Control : Untreated DNA

$$ \mathrm{Enzyme}\ \mathrm{activity} = \frac{\left[{A}_{260}(test)-{A}_{260}(control)\right]\times 20}{0.001\times 15} $$

### Protease treatment and heat inactivation of hPLSCR1

hPLSCR1 was either heat inactivated by incubating at 65 °C for 15 min or treated with trypsin for 60 min at 37 °C and then used for the nuclease assays and Kunitz assays as described earlier. Mock treated hPLSCR1 was used as a positive control.

### Effect of various parameters on nuclease activity

Nuclease assays were performed as described earlier with varying incubation times such as 5, 10, 15, 30, 60, 90 min respectively and were analyzed on a 1 % agarose gel stained with ethidium bromide. Nuclease assay was also performed at various enzyme concentrations and visualized on a 1 % agarose gel as described earlier. Various substrates such as RNA, linear dsDNA, ssDNA and plasmid DNA were used for nuclease assay to identify the specificity of nuclease activity of hPLSCR1. The effect of temperature on nuclease activity was observed by performing assays at 25 °C, 30 °C, 37 °C, 40 °C, 45 °C and 50 °C and incubated for 60 min for nuclease assay or 15 min for Kunitz assay. Nuclease assays were performed at various pH ranging from 4.5 to 9.5 with pH 8 as the reference pH and were quantified using Kunitz assay or visualized on 1 % agarose gel as described earlier. Effect of metal ions on nuclease activity was studied by performing nuclease assays using an assay buffer without MgCl_2_ and adding 10 mM of MgCl_2_, 10 mM of CaCl_2_ and 10 mM of ZnCl_2_ respectively. Dose dependent studies were also performed for Ca^2+^, Mg^2+^ and Zn^2+^ (5 mM, 10 mM, 15 mM, and 20 mM). Nuclease assays and Kunitz assays were set up in the presence and absence of 4 mM of different amino acid modifiers namely, N-ethylmaleimide (NEM), 4-(2-aminoethyl) benzenesulfonyl fluoride hydrochloride (AEBSF), diethyl pyrocarbonate (DEPC), phenyl glyoxal (PG). The reaction mixtures were incubated at 37 °C for 60 min and analyzed on a 1 % agarose gel. Dose dependent Kunitz assays were performed for hPLSCR1 with 2 mM, 4 mM, 8 mM and 16 mM of DEPC.

### Circular Dichroism studies

Circular Dichroism (CD) studies were performed with a JASCO J-810 spectropolarimeter (Jasco, MD, USA). Far UV-CD spectra were recorded at 25 °C with a thermostat cell holder. 10 μM of protein in assay buffer was used for the scan with increasing concentrations of MgCl_2_. Samples were scanned using 1 mm path length cuvettes from 250 to 190 nm.

### Statistical analysis

Data represented was statistically analyzed using Student’s *t*-test and *p* < 0.01 was considered to be significant.

## Results

### Overexpression and purification of recombinant hPLSCR1

**Fig. 1:**
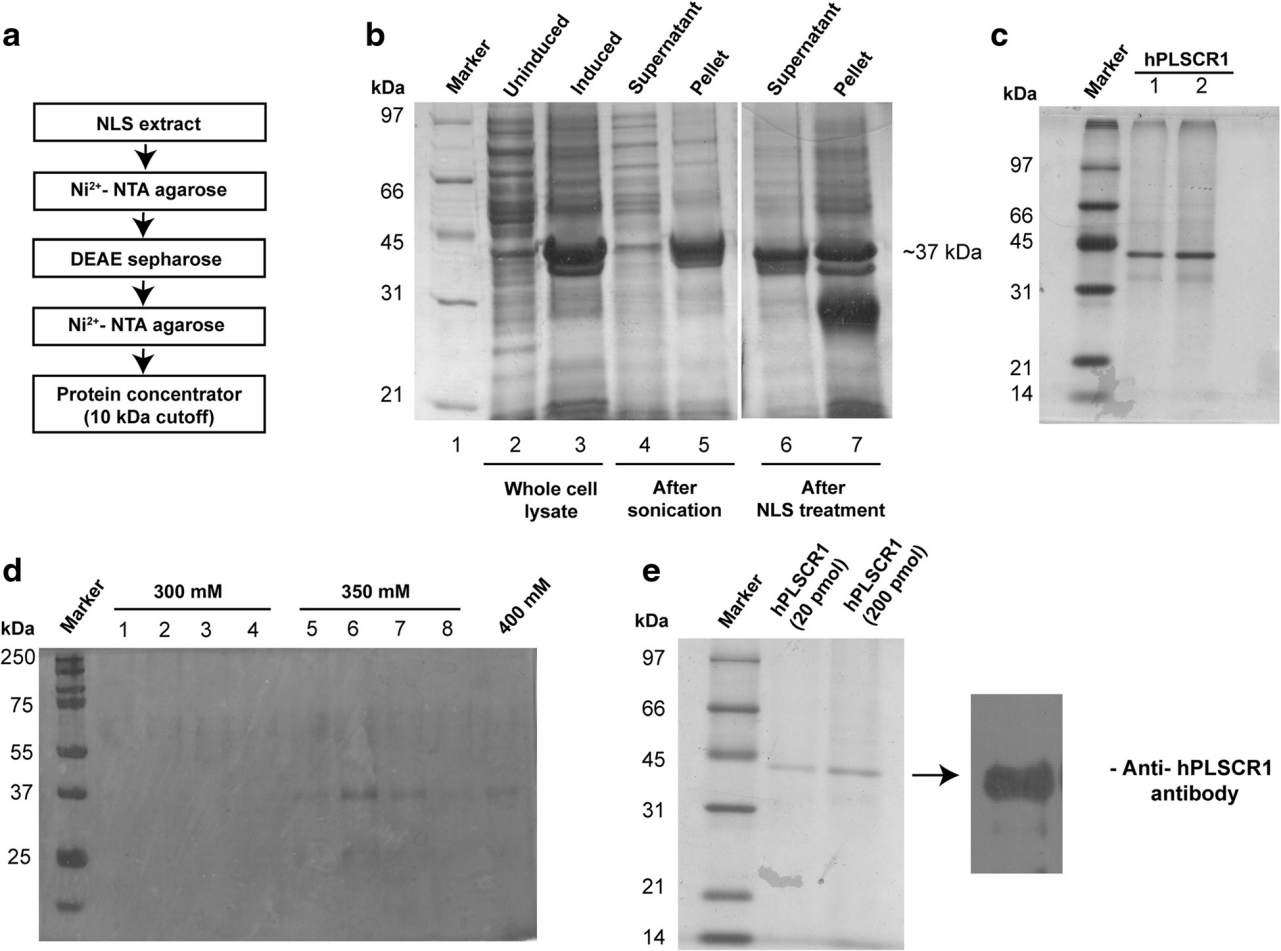
Overexpression and purification of recombinant hPLSCR1 in *E. coli* BL21 (DE3). **a** Schematic showing the steps involved in recombinant purification of hPLSCR1 **b** Coomassie stained SDS-PAGE gel showing hPLSCR1 at each stage of purification: Recombinant hPLSCR1 was overexpressed in *E. coli* BL21 (DE3) and seen as an intense band in lane 3, compared to the uninduced cells (Lane 2). The cells were then lysed by sonication. Most of the overexpressed protein was extracted as inclusion bodies (Lane 5) with low amounts in the soluble fraction (Lane 4). N-Lauroylsarcosine recovered active protein from the inclusion bodies into the soluble fraction (Lane 6). **c** Silver stained SDS-PAGE gel showing purified hPLSCR1: The N-LS recovered protein was purified to homogeneity by Ni^2+^ - NTA chromatography and the elutes were loaded on to 12 % SDS PAGE. **d** DEAE anion exchange chromatography was performed for the elutes from Ni^2+^-NTA chromatography and then eluted with a NaCl gradient. hPLSCR1 eluted in 350 mM NaCl (Lane 5–8). **e** The 350 mM DEAE fractions containing hPLSCR1 were pooled and again passed through Ni^2+^-NTA resin and eluted with 250 mM imidazole. The eluted samples were then concentrated and estimated. Left panel shows the silver stained SDS-PAGE gel showing 20 pmol and 200 pmol of purified hPLSCR1. Western blotting was performed for the purified hPLSCR1, where a specific monoclonal antibody against hPLSCR1 was used and visualized by chemiluminescence as shown in the right panel

Recombinant hPLSCR1 was overexpressed in *E. coli *BL21 (DE3) and purified to homogeneity as described in the literature [34]. The purification steps are shown in the schematic (Fig. 1a). Briefly, hPLSCR1-pET28a (+) was transformed in *E. coli* BL21 (DE3) cells and induced with IPTG for overexpression. Upon sonication, the hPLSCR1 localized as inclusion bodies (IB) (Lane 5, Fig. 1b) with little or no protein in the soluble fraction (Lane 4, Fig. 1b). The IBs were treated with N-Lauroylsarcosine (N-LS), which recovered active protein from IBs to the soluble fraction (Lane 6, Fig. 1b). The N-LS was removed by pulse dialysis as described earlier [34] and N-LS removed protein was purified by His tag - Ni^2+^-NTA chromatography (Fig. 1c). The eluted fractions were dialyzed against purification buffer (20 mM Tris–HCl (pH-7.4), 200 mM NaCl) to remove imidazole and then passed through DEAE sepharose column. hPLSCR1 was eluted at 350 mM of NaCl and the eluted fractions were then loaded again onto a Ni^2+^-NTA column (Fig. 1d). In order to enhance the purity, His-tag purification was repeated. Finally, the protein was concentrated using centrifugal filters and protein content was estimated by BCA method. To confirm the purity, 200 pmol (10 times higher than used in nuclease assay) of the purified protein was loaded on a 12 % SDS-PAGE (silver staining). A single band corresponding to 37 kDa clearly showed that the purified protein does not have other contaminant proteins (Fig. 1e, left panel). The purified protein was further confirmed by western blotting using anti-hPLSCR1 antibody. A single band corresponding to hPLSCR1 was visualized confirming that the purified recombinant protein was indeed hPLSCR1 (Fig. 1e, right panel).

### Decatenation assay for topo IIα in the presence of hPLSCR1

**Fig. 2:**
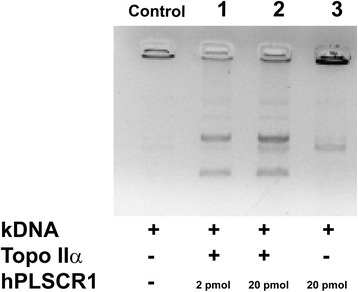
Topo IIα decatenation assay in presence of hPLSCR1. Decatenation assay was performed for topoisomerase IIα in the presence of hPLSCR1 as described in 'Methods'. 200 ng of kinetoplast DNA (kDNA) is treated with 1 U of topo IIα for 15 min at 37 °C and was then visualized on a 1 % agarose gel. Control denotes negative control where only kDNA is present. Lane 1 and lane 2 corresponds to the decatenation assay performed with topo IIα pre-treated with 2 pmol of hPLSCR1 and 20 pmol of hPLSCR1 respectively. Lane 3 corresponds to the decatenation assay in the absence of topo IIα where only hPLSCR1 was added to kDNA

hPLSCR1 is known to interact and enhance the decatenation activity of topo IIα. hPLSCR1 could enhance the decatenation activity of topo IIα by either aiding in unwinding and cleaving of the substrate DNA bound to topo IIα or could have independent nuclease properties which could compliment the decatenation activity of topo IIα. To understand the characteristics of this interaction, we performed decatenation assay for topo IIα in the presence of hPLSCR1. When low amount (2 pmol) of hPLSCR1 was added, decatenation activity of topo IIα was enhanced (Lane 1, Fig. 2) but when the hPLSCR1 concentration was increased (20 pmol), apart from enhancement of decatenation activity, a new distinct band was observed (Lane 2, Fig. 2). This was further confirmed by performing the decatenation assay in the absence of topo IIα (Lane 3, Fig. 2). Results showed presence of the distinct band when only hPLSCR1 was added, thereby revealing that hPLSCR1 processes kDNA independently of topo IIα and could possess nuclease properties (Fig. 2).

### Nuclease activity of hPLSCR1

**Fig. 3:**
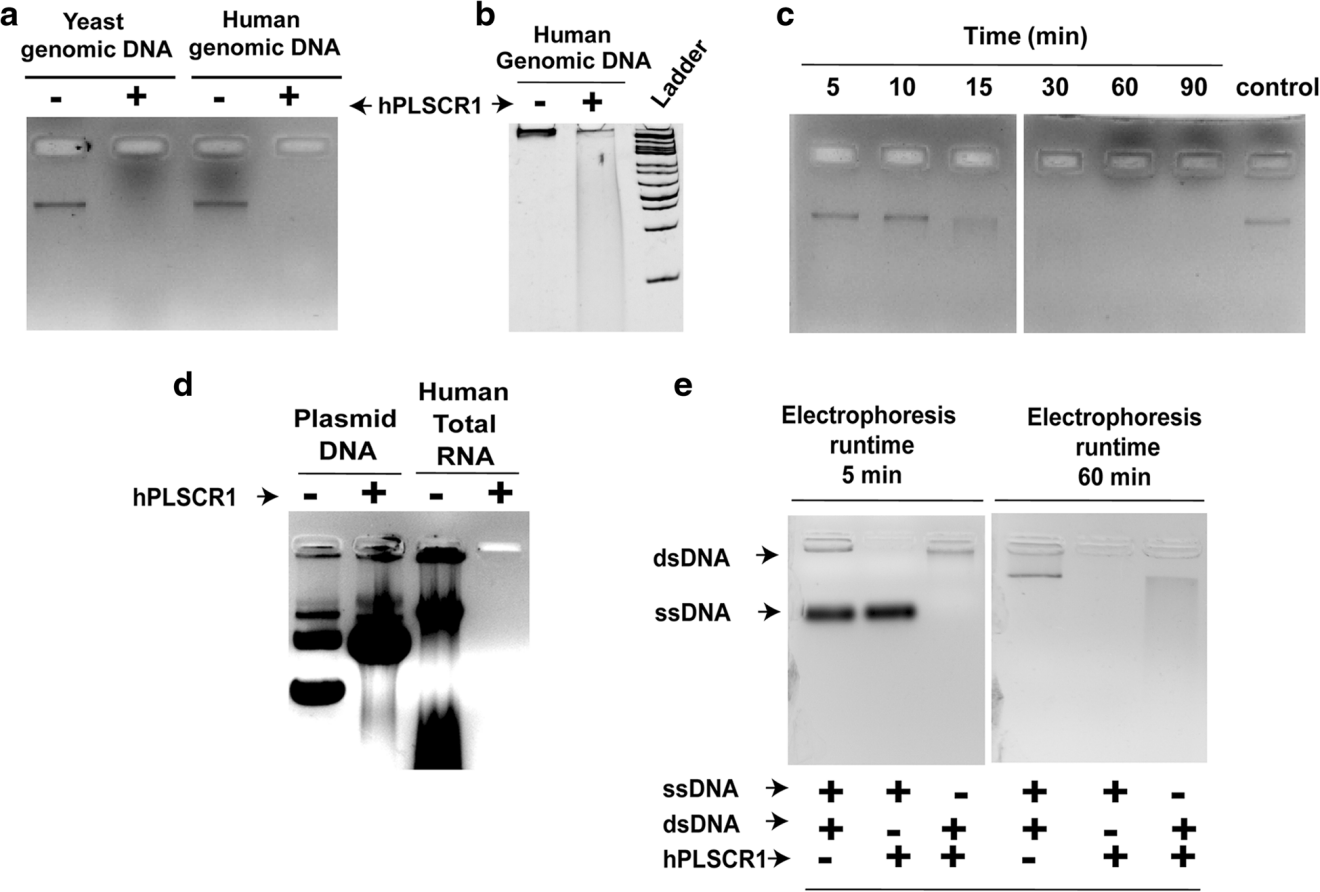
Nuclease properties of hPLSCR1. **a** Nuclease assay for hPLSCR1 with yeast and human genomic DNA (gel assay): Nuclease assay was performed as described in ‘Methods’ section. 20 pmol of hPLSCR1 (enzyme) was treated with 200 ng of yeast and human genomic DNA (substrate) in assay buffer at 37 °C for 60 min and visualized on 1 % agarose gel with ethidium bromide. **b** Native PAGE for visualization of nuclease activity (gel assay): 200 ng of human genomic DNA was incubated with hPLSCR1 at 37 °C for 60 min and visualized on a 12 % native PAGE stained with 0.5 μg/ml ethidium bromide. ‘Ladder’ denotes 1 kb ladder. **c** Time dependent nuclease activity of hPLSCR1 (gel assay): hPLSCR1 (20 pmol) was incubated with human genomic DNA (200 ng) at 37 °C for 5, 10, 15, 30, 60, 90 min along with a no-enzyme control (control) and visualized on 1 % agarose gel with ethidium bromide. **d** Effect of substrates on nuclease activity (gel assay): Nuclease assays were performed with 200 ng of plasmid DNA and total RNA as substrates along with a no-enzyme control visualized on a 1 % agarose gel. **e** Nuclease activity of hPLSCR1 with ssDNA (gel assay): Nuclease assays were performed with 200 ng of a random ssDNA (43 bases) and dsDNA (human genomic DNA) along with a no-enzyme control containing both the substrates and visualized on a 2 % agarose gel

In-vitro nuclease assays were done by incubating 20 pmol of hPLSCR1 with 200 ng of genomic DNA as substrate for 60 min at 37 °C. Yeast genomic DNA and human genomic DNA were used as substrates for the nuclease reaction (Fig. 3a). Presence of nuclease activity was confirmed by the disappearance of the band implying that the DNA was completely degraded when treated with hPLSCR1 as visualized on a 1 % agarose gel stained with ethidium bromide; 12 % native PAGE was also used to better resolve the products formed during the nuclease reaction (Fig. 3b). hPLSCR1 incubated with genomic DNA migrated with a retarded smear, thus confirming the nuclease activity of hPLSCR1. Nuclease reaction was then performed for different time periods (5, 10, 15, 30, 60, 90 min) at 37 °C. Time course study revealed that the degradation of DNA happens as early as 10 min with the complete degradation at 60 min for 200 ng of DNA (Fig. 3c). Nuclease assay was also performed with total RNA, plasmid DNA and single stranded DNA (ssDNA) as substrates. hPLSCR1 exhibited nuclease activity in dsDNA, RNA and but no nuclease activity was observed when treated with ssDNA (Fig. 3d and e). Interestingly, hPLSCR1 nicks the plasmid DNA as evident from the figure as a single band is observed when plasmid DNA is treated with hPLSCR1 (Fig. 3d). To further confirm that the purified protein is devoid of other contaminating proteins (negative control), pET 28a (+) vector without the insert was transformed in *E. coli* and the purification protocol was repeated as described earlier. SDS-PAGE analysis did not reveal any contaminating proteins in the negative control and the resulting sample did not have any nuclease activity (data not shown). This revealed that the recombinant hPLSCR1 is not contaminated with host proteins, which could contribute to the nuclease activity of hPLSCR1.

**Fig. 4:**
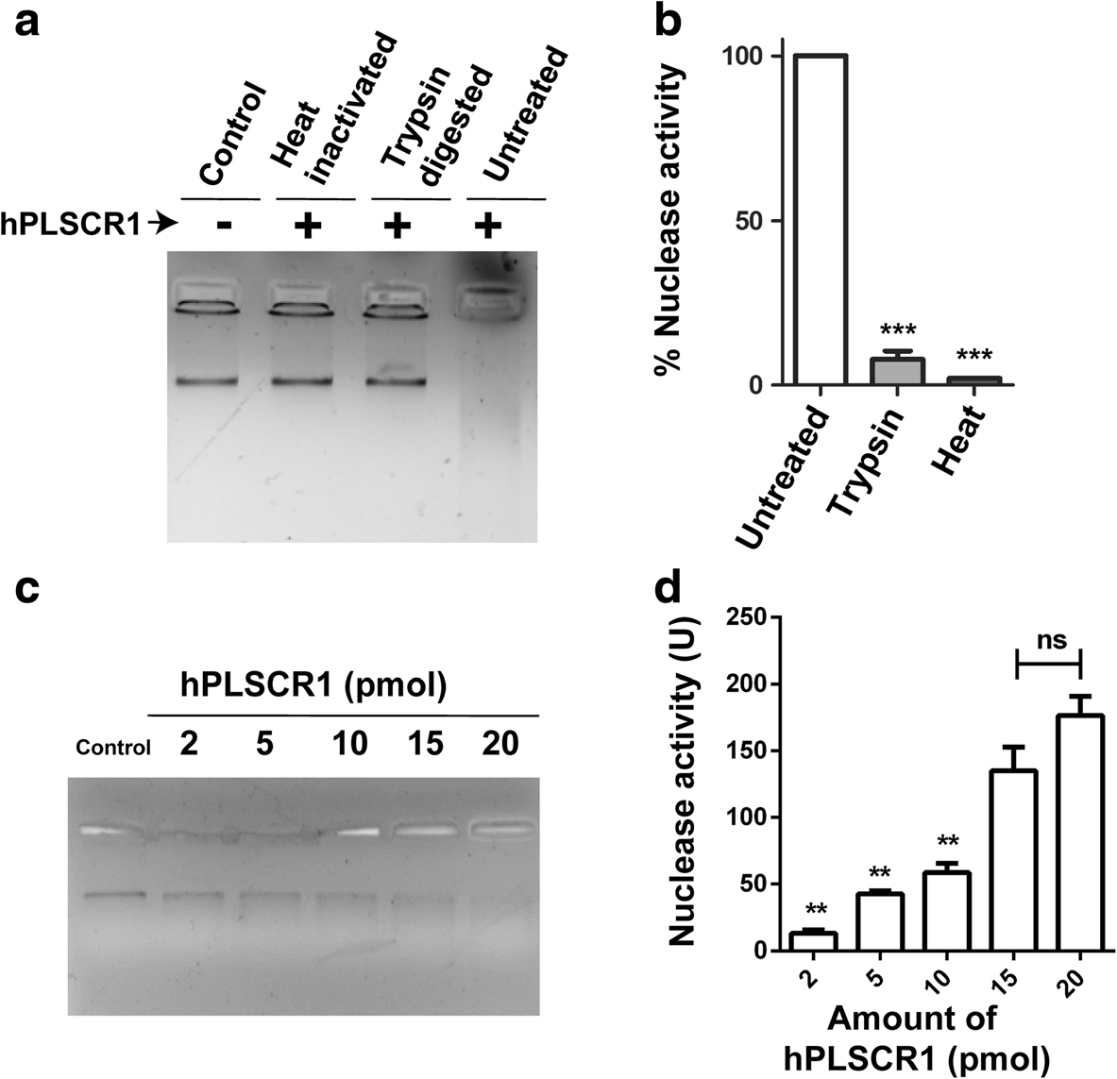
Nuclease activity is protein mediated. **a**, **b** Nuclease assay upon protein inactivation: gel assay (**a**) and Kunitz assay (**b**) were performed with heat-inactivated and trypsin digested hPLSCR1 (20 pmol) along with native hPLSCR1 (untreated) and a no enzyme control (control). **c** Effect of hPLSCR1 concentration on nuclease activity (gel assay): 200 ng of human genomic DNA was incubated with various concentrations of hPLSCR1 (2 pmol, 5 pmol, 10 pmol, 15 pmol and 20 pmol) at 37 °C for 60 min and visualized on a 1 % agarose gel stained with 0.5 μg/ml ethidium bromide. ‘Control’ denotes negative control. **d** Kunitz assay was performed for varying concentrations of hPLSCR1 as described above. * denotes statistical significance at *p* < 0.05, ** denotes statistical significance at *p* < 0.005; ns – not significant. Experiments were performed in triplicates and error bars denote standard deviation

To reconfirm this, nuclease assay and Kunitz assay (as described in 'Methods') was performed with heat inactivated and trypsin treated hPLSCR1 and results confirmed that nuclease activity was protein mediated as the genomic DNA was not degraded when trypsin digested or heat inactivated protein was used for the nuclease reaction (Fig. 4a and b). Nuclease assay and Kunitz assay was performed with increasing hPLSCR1 concentrations (2 pmol, 5 pmol, 10 pmol, 15 pmol and 20 pmol). Results clearly explained that there is a dose dependent increase in the nuclease activity confirming that the nuclease reaction is dependent on the concentration of hPLSCR1 (Fig. 4c and d).

### Parameters affecting nuclease activity

**Fig. 5:**
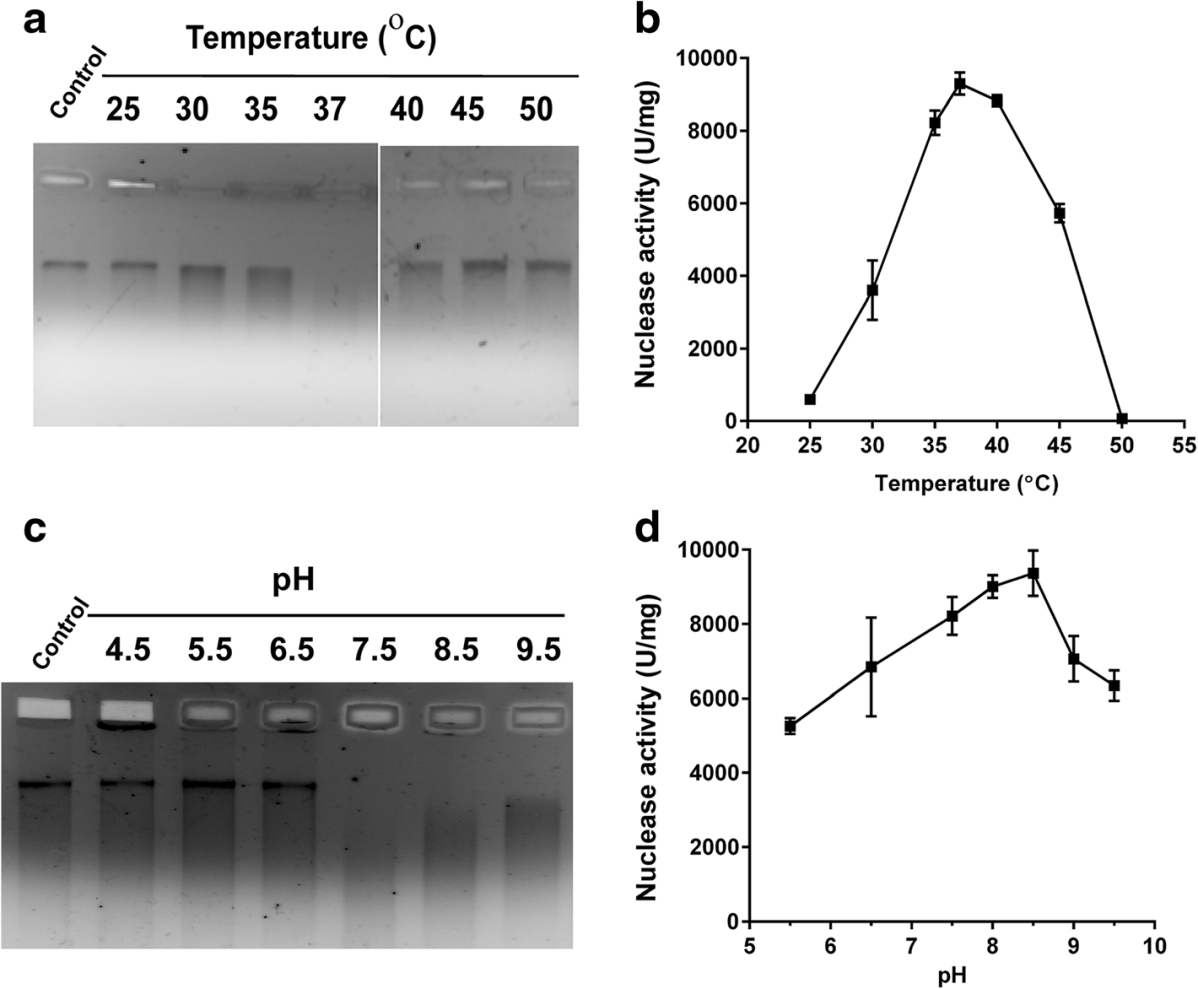
Effect of temperature and pH on nuclease activity of hPLSCR1. **a** Temperature effect on nuclease activity (gel assay): hPLSCR1(20 pmol) was incubated with human genomic DNA (200 ng) for 60 min at different temperatures such as 25, 30, 37, 40, 45, 50 °C along with a no-enzyme control ‘Control’ and were visualized on a 1 % agarose gel. **b** Temperature effect on nuclease activity (Kunitz assay): Kunitz assay was performed as described in ‘Methods’ section. The calculated specific activity values from Kunitz assay at various temperatures (25, 30, 37, 40, 45 °C) were plotted. **c** pH effect on nuclease activity (gel assay): hPLSCR1 (20 pmol) was incubated with human genomic DNA (200 ng) for 60 min at different pH such as 4.5, 5.5, 6.5, 7.5, 8.5, 9.0, 9.5 along with no-enzyme control named ‘Control’ and visualized on a 1 % agarose gel. **d** pH effect on nuclease activity (Kunitz assay): Kunitz assay was performed as described under ‘Methods’ section. The calculated specific activity values from Kunitz assay at various pH (6.5, 7.5 8.0, 8.5, 9.0, 9.5) were plotted. Experiments were performed at-least three independent times and error bars denote standard deviation

We tested the effect of pH, temperature, cofactors and protein modifying reagents to characterize the nuclease function of hPLSCR1. hPLSCR1 exhibited maximum activity of 9297.3 U/mg at 37 °C and the activity reduced significantly beyond 45 °C, which was confirmed by gel assay and Kunitz assay (Fig. 5a and b). A decrease in enzyme activity of 96 % and 40 % was observed when incubated at 25 °C and 45 °C respectively. In contrast to temperature studies, hPLSCR1 was found to be active over a range of pH 6.0–9.0 with maximum activity at 8.5 (Fig. 5c and d).

**Fig. 6:**
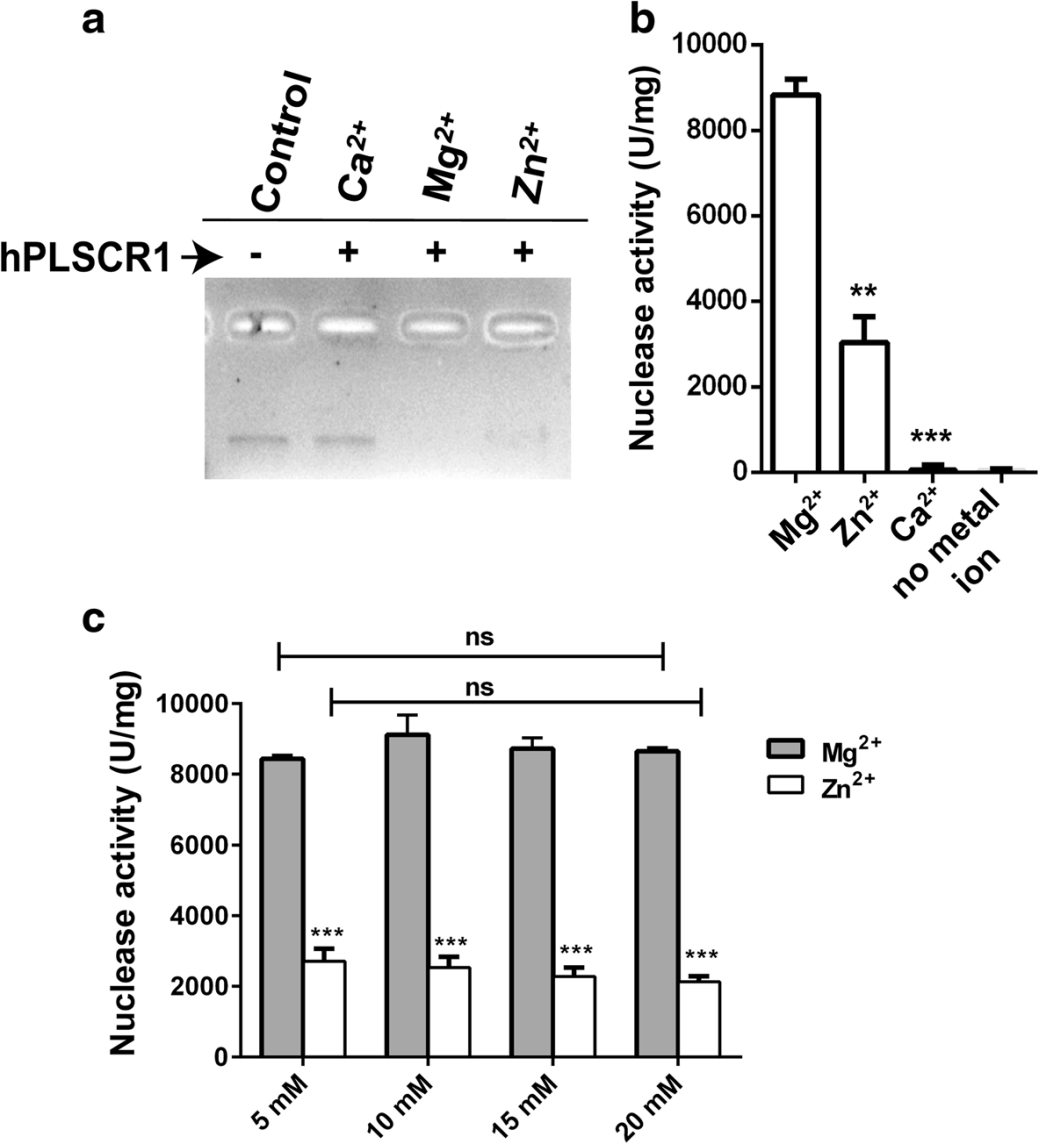
Effect of metal ions on nuclease activity of hPLSCR1. **a** Gel assay: hPLSCR1 was incubated with human genomic DNA in the presence of 10 mM of CaCl_2_, MgCl_2_ and ZnCl_2_ at 37 °C for 60 min and visualized on a 1 % agarose gel. **b** Metal ions effect on nuclease activity (Kunitz assay): Nuclease activity was calculated from Kunitz assay done for 10 mM of CaCl_2_, MgCl_2_ and ZnCl_2_ or in the absence of metal ions. **c** Dose dependence of MgCl_2_ and ZnCl_2_ on nuclease activity of hPLSCR1. Kunitz assays were performed with 5 mM, 10 mM, 15 mM and 20 mM of MgCl_2_ and ZnCl_2_. * denotes statistical significance at *p* < 0.05, ** denotes statistical significance at *p* < 0.005; *** denotes statistical significance at *p* < 0.0005; ns – not significant. Experiments were performed in triplicates and error bars denote standard deviation

Nuclease reactions were set up in the presence of different metal ions like Ca^2+^, Mg^2+^ and Zn^2+^. hPLSCR1 exhibited a maximum activity of 8828.83 U/mg in the presence of Mg^2+^, whereas a 70 % decrease in activity was observed with Zn^2+^ and no activity was observed in the presence of Ca^2+^ (Fig. 6a and b). Different concentrations (5 mM, 10 mM, 15 mM and 20 mM) of Ca^2+^, Mg^2+^ and Zn^2+^ were tested for nuclease activity. Calcium did not exhibit activity at any concentration (Data not shown) whereas there was no significant change in nuclease activity values with increasing concentrations of Mg^2+^ and Zn^2+^ (Fig. 6c).

### Effect of protein modifying reagents on nuclease activity

**Fig. 7:**
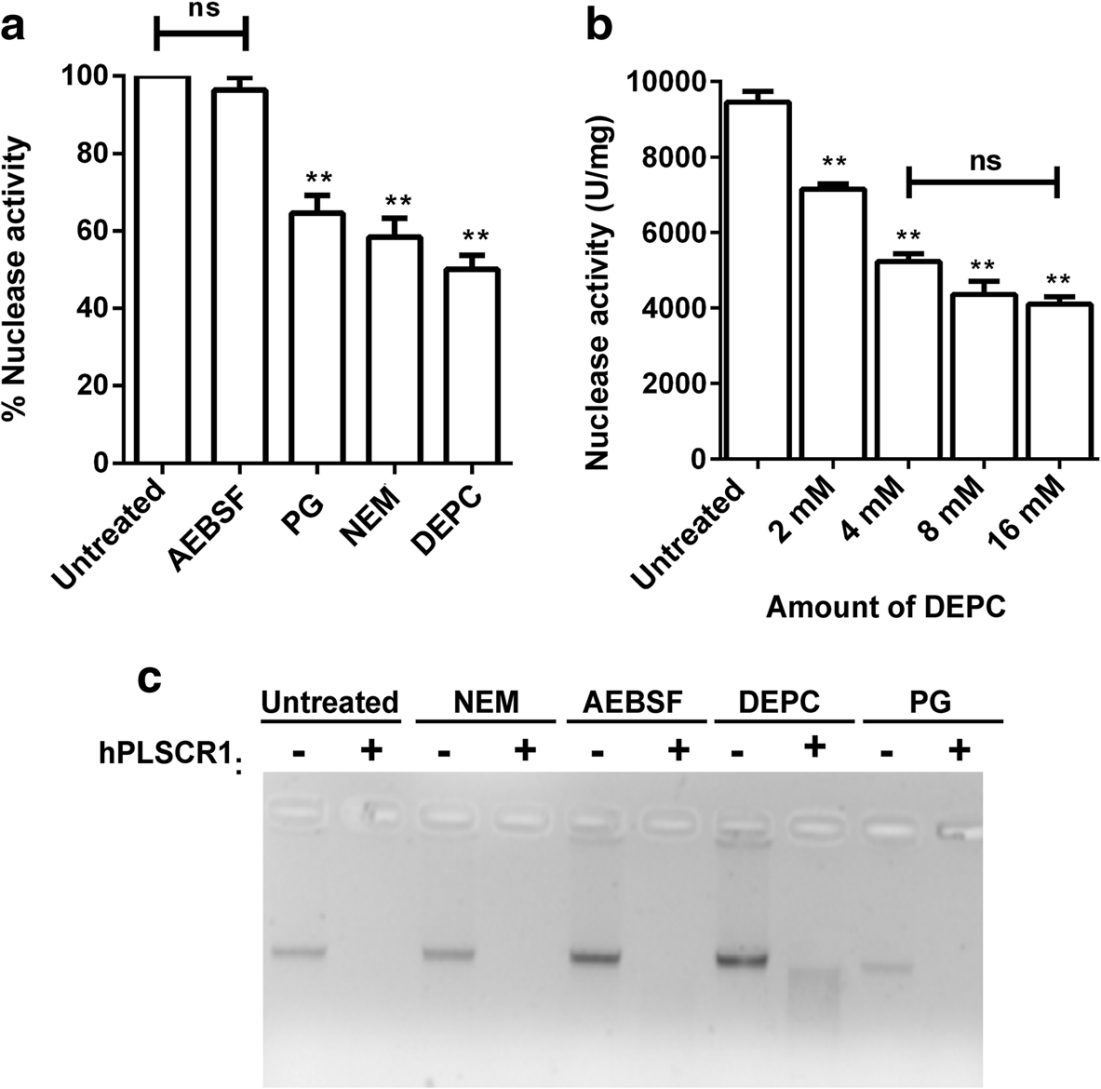
Effect of amino acid modifying reagents on nuclease activity of hPLSCR1. **a** Kunitz assay: Kunitz assay was used to calculate specific activity of hPLSCR1 (20 pmol) treated with 4 mM of each of the amino acid modifiers including NEM, PG, DEPC, AEBSF along with native hPLSCR1 (untreated). Graph shows the percentage activity of modifier treated hPLSCR1 compared to the untreated hPLSCR1. **b** DEPC dose dependence studies on nuclease activity: Kunitz assays were used to calculate specific activity of hPLSCR1 treated with an increasing concentration of DEPC (2 mM, 4 mM, 8 mM, and 16 mM) and plotted. **c** Effect of protein modifying reagents on nuclease activity (gel assay): Nuclease assays for hPLSCR1 treated with 4 mM of NEM, AEBSF, DEPC, and PG at 37 °C for 60 min were visualized in 1 % agarose gel. * denotes statistical significance at *p* < 0.05, ** denotes statistical significance at *p* < 0.005; ns – not significant. Experiments were performed in triplicates and error bars denote standard deviation

hPLSCR1 was treated with 4 mM of amino acid modifiers – NEM (cysteine modifier), DEPC (histidine modifier), AEBSF (serine modifier) and PG (arginine modifier) and checked for nuclease activity (Fig. 7a). Among all those tested, DEPC treated hPLSCR1 showed 51 % inhibition (3935.24 U/mg) whereas PG and NEM showed 40 and 45 % inhibition when compared to the untreated hPLSCR1. This suggests that histidine, cysteine and arginine residues are crucial for nuclease activity. AEBSF did not inhibit the nuclease activity indicating that serine does not affect the nuclease activity. A dose dependent inhibition was observed when hPLSCR1 was treated with increasing concentrations of DEPC (Fig. 7b). When the nuclease assay was performed for an incubation time of 60 min, DEPC exhibited more inhibition than any other reagent, which is in accordance with specific activity values (Fig. 7c). In the absence of DEPC, complete degradation of DNA similar to untreated sample was observed. In the presence of DEPC, the reaction was inhibited as seen by the smeared DNA in the DEPC lane compared to the disappearance of band in the other amino acid modifiers. Based on Kunitz and gel assay results, it was found that DEPC showed the most inhibition among the tested amino acid modifiers.

### Role of histidine residues in nuclease activity of hPLSCR1

**Fig. 8:**
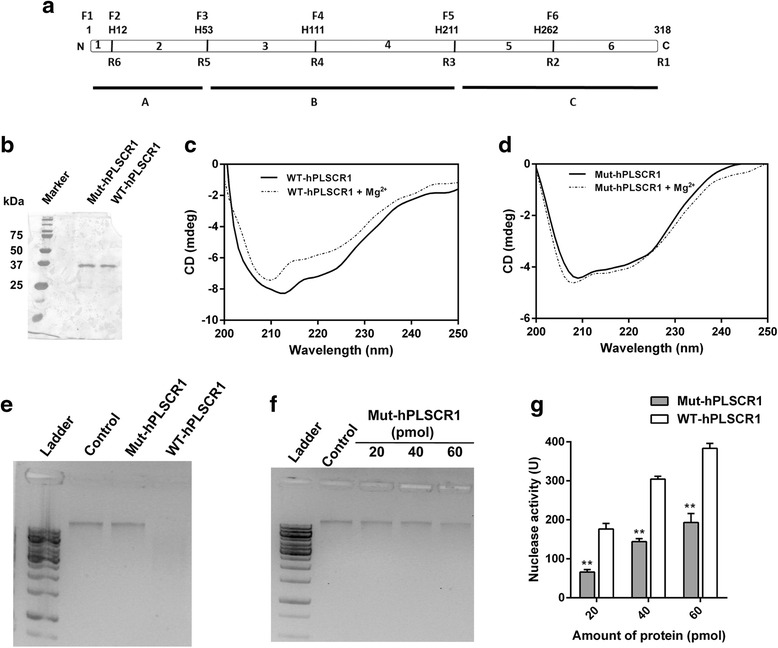
Histidine residues are essential for nuclease activity of hPLSCR1. **a** Schematic showing the position of five histidine residues in hPLSCR1. F1-F6 and R1-R6 were the forward primers and reverse primers used for generation of mutations respectively. A detailed methodology for generation of point mutants is described in 'Methods' section. **b** Silver stained SDS-PAGE gel showing 20 pmol of Mut-hPLSCR1 along with WT-hPLSCR1 purified to homogeneity. Circular Dichroism studies for WT-hPLSCR1 (**c**) and Mut-hPLSCR1 (**d**) in the presence and absence of 3 mM MgCl_2_ were shown. **e** Nuclease assay for Mut-hPLSCR1 (gel assay): Gel assay was performed for Mut-hPLSCR1 and WT-hPLSCR1 as described in ‘Methods’ section and visualized on a 1 % agarose gel. **f** Dose dependence of Mut-hPLSCR1 by gel assay: Nuclease assay was performed at increasing concentration of Mut-hPLSCR1 (20 pmol, 40 pmol, 60 pmol) and visualized on a 1 % agarose gel. **g** Dose dependence studies for Mut-hPLSCR1 by Kunitz assay: Kunitz assay was performed to quantify the nuclease activity of increasing concentrations of Mut-hPLSCR1 (20 pmol, 40 pmol, 60 pmol) (grey bars) and compared with the dose dependent nuclease activity of WT-hPLSCR1 (white bars). ** shows statistical significance at *p* < 0.005; ns- not significant. Experiments were repeated at least three independent times and the error bars denote standard deviation

Amino acid modifications of hPLSCR1 revealed that DEPC, majorly a histidine modifier showed maximum inhibition of nuclease activity. hPLSCR1 has five histidines (Fig. 8a) and all the five histidines (H12, H53, H111, H211, H262) were mutated to alanine and the clone was named as Mut-hPLSCR1. Mut-hPLSCR1 was then cloned, overexpressed, purified and analyzed on a 12 % SDS-PAGE using silver staining. A single band corresponding to 37 kDa was observed confirming the presence of Mut-hPLSCR1 (Fig. 8b). In order to confirm that the point mutations did not destabilize the structure of the protein, CD studies were performed. Results revealed that Mut-hPLSCR1 retained the alpha helical signature that was observed in wild type (WT-hPLSCR1) thus showing that the mutant protein is in its native form (Fig. 8c and d). Nuclease assay was performed with Mut-hPLSCR1 and WT-hPLSCR1. Results revealed that the nuclease activity was relatively very low in Mut-hPLSCR1 compared to WT-hPLSCR1 (Fig. 8e). Nuclease activity by gel assay with increasing concentrations of Mut-hPLSCR1 showed that a negligible nuclease activity even when the concentration was increased by 3 fold (60 pmol) than normal assay conditions (Fig. 8f). In addition, the nuclease activity of Mut-hPLSCR1 was also quantified by Kunitz assays. At low concentrations (2-10 pmol), nuclease activity was not observed for Mut-hPLSCR1 and at higher concentrations (20-60 pmol), 60 % loss in nuclease activity of Mut-hPLSCR1 compared to WT-hPLSCR1 (Fig. 8g). The reminiscent nuclease activity might be due to several other factors, which need to be further investigated. These results clearly explain that the histidine residues are vital for nuclease activity of hPLSCR1.

## Discussion

hPLSCR1 is a multifunctional protein involved in several key cellular pathways such as cell cycle, apoptosis, Ca^2+^ homeostasis. In this study, we demonstrated that hPLSCR1 possesses novel function of nuclease activity for the first time. Finding the physiological implications of the nuclease activity of hPLSCR1 could pave way to identify new roles for hPLSCR1 in several cellular processes. Even though hPLSCR1 was reported to enhance the decatenation activity of topo IIα, the underlying mechanism is not yet deciphered. hPLSCR1 could either (i) alter the structure of topo IIα thereby leading to an increase in the catalytic activity; (ii) help in unwinding or cleaving of the DNA substrate bound to topo IIα; (iii) possess independent nuclease properties that aid in decatenation activity of topo IIα. To investigate this, the decatenation assay was performed with topo IIα pretreated with 2 pmol and 20 pmol of hPLSCR1. At low amounts of hPLSCR1, we observed enhancement of decatenation activity similar to previous report [25]. When 20 pmol of hPLSCR1 was used in the decatenation assay, a distinct band was observed along with the decatenation products of topo IIα. The appearance of this unique band was further confirmed by performing the decatenation assay with hPLSCR1 in the absence of topo IIα explaining that apart from enhancing the decatenation activity of topoisomerases, hPLSCR1 could independently possess nuclease properties which could aid in the enhancing the catalytic activity of topo IIα. Topo IIα is known to interact with CAD nuclease and is involved in chromatic condensation. The CAD nuclease apart from digesting DNA during apoptotic execution, it also interacts with topo IIα and enhances the decatenation activity of topo IIα in vitro [36]. In another report, topo IIβ interacts with an extracellular nuclease in mammalian spermatozoa and the interaction enhances the decatenation activity of topo IIβ [37]. Similarly, we believed that hPLSCR1 could have nuclease properties, which could be the reason for enhancement of decatenation activity of topo IIα in vitro. To support this hypothesis, sequence alignment of hPLSCR1 with known nucleases with bioinformatics tools revealed some key findings. PRD of hPLSCR1 has vast similarities with the PRD of TREX1, a mammalian member of DnaQ-like 3′–5′ exonuclease family [38]. TREX1 exonuclease activity is supported by a metal ion catalyzed phosphoryl transfer mechanism. TREX1 constitutes about 45 prolines and its PRD contains about 6 prolines, which are involved in interactions with various protein complexes [38, 39]. Sequence analysis showed that about 16 out of 29 prolines in PRD of hPLSCR1 were aligned and 4 prolines of TREX1 PRD exactly corresponded with PRD of hPLSCR1 (Data not shown). The Mg^2+^ binding site (^176^**A**T**ED**M**D**CLT^184^) in human FLAP endonuclease I (FEN1), a protein involved in DNA replication and repair was also conserved in hPLSCR1 (^142^**A**A**ED**T**D**CCT^150^). The bold characters in the sequence indicate the residues involved in Mg^2+^ binding in FEN1, which are conserved in hPLSCR1. Hence, these striking similarities along with various other factors in the literature such as Mg^2+^ binding, up regulation by interferons, involvement during apoptosis, interaction and enhancement of decatenation activity of topo IIα and processing of kDNA even in absence of topo IIα intrigued us to elucidate the unique property of hPLSCR1 as a nuclease.

Purified recombinant hPLSCR1 exhibited a dose dependent nuclease activity when incubated with yeast genomic DNA and human genomic DNA at 37 °C. Overexpression and purification was repeated for *E. coli* cells transformed with pET 28a (+) vector without any insert (vector-purified protein). No distinct bands were revealed in SDS-PAGE analysis of the vector-purified protein and the samples did not show any nuclease activity (data not shown). A random 37 kDa protein that was purified by the same protocol as mentioned in ‘Methods’ was also tested as a negative control to eradicate the possibilities of a co-purified contaminating host nuclease because of the purification protocol (data not shown). Absence of nuclease activity in vector-purified sample and in random protein clearly states that the nuclease activity is borne only by hPLSCR1 and not by other host contaminating nucleases. hPLSCR1 was active at temperatures between 25 and 45 °C (Fig. 5a), where a temperature of 37 °C was found to be optimum for its activity, which was similar for many other human nucleases such as TREX1, FLAP endonuclease 1 (FEN 1) and DNase 1. The optimal pH for nuclease activity was found to be between pH 8.0 and 9.0 (Fig. 5b) suggesting that hPLSCR1 probably acts as an alkaline nuclease. Most of the nonspecific nucleases such as TREX1 and FEN1 had highest activity at 7.5 and 8.0 respectively [38, 40]. This was further confirmed by assaying nuclease activity with various substrates such as ssDNA, dsDNA and RNA. hPLSCR1 exhibited nuclease activity towards dsDNA and RNA but not ssDNA. hPLSCR1 exhibited nicking activity towards plasmid DNA which could explain that hPLSCR1 might be a nicking endonuclease which needs to be further investigated. TREX-1 digests ssDNA and dsDNA with mismatched 3′ termini [41]. These findings suggest that hPLSCR1 is a non-specific alkaline nuclease.

Majority of nucleases require metal ions such as Mg^2+^, Ca^2+^ and Zn^2+^ for their activity. Metal ions in nucleases are found to play a dual role: (i) enhancing the affinity of the substrate to the enzyme which could be sequence or structure specific and (ii) directly involved in catalysis where the phosphate oxygen bond is cleaved [42]. To verify the specificity of metal ions for nuclease activity of hPLSCR1, assays were performed with Ca^2+^, Zn^2+^ and Mg^2+^ (Fig. 6). Ca^2+^ did not induce nuclease activity, which was astonishing as Ca^2+^ binding is vital for the PL translocation by hPLSCR1 across the lipid bilayer. It was found that Mg^2+^ and Zn^2+^ stimulated nuclease activity and might possibly play a role in stabilizing the enzyme structure apart from catalysis. hPLSCR1 has an EF hand like Ca^2+^ binding motif and point mutations in the D275 resulted in loss of calcium binding and scramblase activity [30]. We also reported that Mg^2+^ binds to the EF hand like Ca^2+^ binding motif in hPLSCR1 (D^273^ – D^284^) [31]. Hence to understand the metal binding properties of hPLSCR1, D275A point mutant of hPLSCR1 was generated and checked for scramblase activity and nuclease activity. The mutant D275A-hPLSCR1 showed a complete loss of scramblase activity, but the nuclease activity was not affected (data not shown). This could possibly explain that Mg^2+^ could have a binding site other than the EF-hand like motif which remains to be investigated. Further studies should be performed to understand the mechanism of Mg^2+^ interaction with hPLSCR1.

Protein modification experiments revealed that histidine, cysteine and arginine residues in hPLSCR1 were important for nuclease activity. Histidines and cysteines are the most commonly occurring active site residues in nucleases. In a similar study, DEPC modification of CAD nuclease inhibited the nuclease activity. CAD nuclease has two histidine residues at its active site that is essential for its nuclease activity. The active site of CAD possesses two histidine residues and DEPC modification inactivated the enzyme [43]. Based on our results, we generated a histidine point mutant (Mut-hPLSCR1) where all the histidine residues in hPLSCR1 were mutated to alanine. Mut-hPLSCR1 showed a threefold decrease in nuclease activity only when assayed at 20-60 pmol and showed statistically insignificant activity at lower concentrations (2-10 pmol). This confirms that the nuclease activity is only by hPLSCR1 and eliminates the possibility of co-purification of a host contaminating nuclease. Histidine could be involved either in the catalytic site or for stabilizing the enzyme substrate complex which needs to be further investigated.

The mechanism and the physiological role of the nuclease property of hPLSCR1 in normal and pathological conditions remain to be investigated. In a recent report, hPLSCR1 was shown to be induced and localized to PM and periplasmic region upon dsDNA transfection in normal immortalized ovarian surface epithelial cells [44]. The physiological relevance of this marked induction is not yet identified. Since it is already well documented that hPLSCR1 is a part of interferon mediated anti-viral defense, the induced hPLSCR1 during dsDNA transfection might have a role as a nuclease in defense against foreign DNA.

## Conclusions

In summary, this is the first report showing a novel Mg^2+^ dependent activity of hPLSCR1. The nuclease activity was strictly metal dependent and could act on dsDNA, RNA but not ssDNA as substrates. DEPC treated hPLSCR1 exhibited reduced nuclease activity and further mutation of histidines resulted in a 60 % loss of activity. Histidines could play a critical role in nuclease activity of hPLSCR1. Further studies are required to ascertain the exact mechanism of nuclease activity, critical residues involved in catalysis, Mg^2+^ binding, substrate binding and the physiological relevance for nuclease activity of hPLSCR1.

## Abbreviations

AEBSF: 4-(2- aminoethyl) benzenesulfonyl fluoride hydrochloride
DEPC: diethyl pyrocarbonate
dsDNA: double stranded DNA
FEN1: Flap endonuclease 1
hPLSCR1: human phospholipid scramblase 1
IFN: interferon
NEM: N-ethyl maleimide
N-LS: N-Lauroylsarcosine
PG: phenyl glyoxal
PL: Phospholipid
PM: plasma membrane
PRD: Proline rich domain
PS: Phosphotidylserine
ssDNA: single stranded DNA
TREX1: 3'-repair exonuclease-1
